# The nursing staff opinion about the continuous quality improvement program of a university hospital

**DOI:** 10.1590/S1679-45082014AO2833

**Published:** 2014

**Authors:** Fernanda Mazzoni da Costa, Rosangela Maria Greco, Elena Bohomol, Cristina Arreguy-Sena, Vitor Luiz Andrade

**Affiliations:** 1 Universidade Federal de Juiz de Fora, Juiz de Fora, MG, Brazil.; 2 Universidade Federal de São Paulo, São Paulo, SP, Brazil.

**Keywords:** Health systems, Health management, Quality management, Working conditions, Nursing staff hospital

## Abstract

**Objective:**

To analyze the nursing staff opinion about the continuous quality improvement program at a University Hospital.

**Methods:**

A descriptive study designed as a case study, analyzing the quality program at a University Hospital, with the opinion of a sample stratified by nursing team category through a self-administered questionnaire, from May to July 2012. The answers were submitted to factor analysis, having the dialectical and historical materialism as the theoretical-methodological reference.

**Results:**

The factor analysis grouped the variables in six factors: working conditions, approval, belongingness, tranquility, interpersonal relations, and private life. With the exception of the factor interpersonal relations, the answers revealed that workers do not have opinion about the proposed questions. Four of the six factors had a predominance of positive answers.

**Conclusion:**

A high percentage of respondents was not aware of the implications of a quality program. The majority believed that the program influenced positively in their working conditions and in the interpersonal relationships at work and agree with the program; however, they did not feel part of the program, and were not at ease to develop these activities. They did not acknowledge the program interfering in their personal life.

## RESUMO

**Objetivo:**

Analisar a opinião dos trabalhadores de enfermagem sobre o programa de melhoria contínua da qualidade em um Hospital Universitário.

**Métodos:**

Estudo descritivo delineado como estudo de caso, cujo objeto de análise foi o programa de qualidade de um Hospital Universitário, com levantamento da opinião de amostra estratificada por categoria da equipe de enfermagem, por meio de questionário autoaplicável, no período de maio a julho de 2012. As respostas foram submetidas à análise fatorial, tendo por referencial teórico-metodológico o materialismo histórico-dialético.

**Resultados:**

A análise fatorial agrupou as variáveis em seis fatores: condições de trabalho, anuência, pertencimento, tranquilidade, relações interpessoais e vida pessoal. Com exceção do fator relações interpessoais, as respostas revelaram que os trabalhadores não têm opinião a respeito das questões propostas. Quatro, entre os seis fatores, tiveram predominância de respostas positivas.

**Conclusão:**

Expressivo percentual dos respondentes não conhecia as implicações de um programa de qualidade. A maioria dos trabalhadores acredita que o programa influencia positivamente em suas condições e nas relações interpessoais no trabalho, e dá anuência ao programa, porém, não se sentiam parte do programa e não tinham tranquilidade para desenvolver suas atividades. Eles também não viam interferência do programa em sua vida pessoal.

## INTRODUCTION

Quality improvement in healthcare contributes to decrease errors and consequently damage to patients. Thus it draws attention of healthcare organizations to quality programs. The advocates of such programs state that by improving the processes, it is possible to advance towards excellence, whose search is endless. Improving the quality of results translates into satisfaction of all stakeholders.^(1,2)^

Achievement of standards, understood as the bases for rising quality, is the main driver of safety efforts,^(3)^ and is encouraged by some organizations, such as the Institute for Healthcare Improvement, United Kingdom National Health Service, Joint Commission on Accreditation of Healthcare Organizations, Agency for Healthcare Research and Quality e United States National Quality Forum.^(4)^

Nevertheless, other studies reported that the companies used refined techniques to manipulate the workers, including the Taylorist approaches of increasing production and generating profit, on which these programs are based.^(5,6)^

In this context, and understanding that all stakeholders are not always those that are relevant to companies' market interests of the companies, this study aimed to consider the perspective of one of these parties - the workers.

## OBJECTIVE

The study aimed to analyze the opinion of nursing professionals about a program of continuous quality improvement developed at a University Hospital.

## METHODS

A descriptive study designed as a case study to analyze the quality program at the *Hospital Universitário de Juiz de Fora*.

The data were collected from May to July 2012 by means of a self-administered questionnaire, previously tested in 10 subjects of the study population, containing 24 statements. The agreement level of participants was expressed by means of statements, in a scale with response amplitude that contemplated five levels.

For sample calculation, the population of 278 nursing professionals was stratified by professional categories based on the variance of responses of the pilot sample of 12 subjects, considering a maximum acceptable error of 5% a confidence interval of 95%, totaling up 82 nursing professionals – in that, 11 were licensed professional nurses (LPN), 55 nurse technicians and 16 registered nurses (RN). The study included workers that delivered care or performed administrative services related to nursing. Workers who did not want to take part and who did not sign the consent form were excluded. A safety margin was considered for replacement of sample losses.

The software Statistical Package for the Social Sciences (SPSS), version 14.0, was used to process the responses submitted to factorial analysis.

The Bartlett test of sphericity was 1004.348, and the Kaiser-Meyer-Olkim test had a value of 0.820, indicating that the sample was adequate for the proposed technique. The cases that did not respond to some statements were excluded; the program took into account 72 answers.

Historical and dialectical materialism was adopted as the theoretical-methodological reference.^(7)^

The investigation was approved by the Research Ethics Committee from the *Universidade Federal de Juiz de Fora*, Juiz de Fora, MG, Brazil (number 262/2010).

## RESULTS

Of the workers who answered the questionnaire, 63% were aged 30-49 years; 78% were women; 53.5% had graduated more than 6 years ago; 54.6% had been working at the organization for longer than 6 years; 68.3% had only one job and 54.9% had only one job.

Factoral analysis of the answers regarding the quality program enabled pooling the variables into six factors: working conditions (evaluates how much the worker believes the quality program interferes in his/her working conditions); approval (investigates agreement with/approval of the quality program); belongingness (quantifies the worker's feeling of belonging to the program); easiness (measures how much easiness the worker believes he/she has to develop his activities); interpersonal relationships (investigates the worker's perception about the interference of quality in relationships) and personal life (investigates the worker's perception about interference of quality in his/her life). The distribution of answers in relation to each factor is shown in table 1.

**Table 1:** Distribution of workers' answers on their opinion in relation to each factor evaluated

Factors	Fully agree n (%)	Partially agree n (%)	Do not have an opinion about it n (%)	Partially disagree n (%)	Fully disagree n (%)	Total n (%)
Factor 1: Working conditions	17 (23.6)	23 (32.0)	15 (20.8)	9 (12.5)	8 (11.1)	72 (100)
Factor 2: Approval	10 (13.9)	21 (29.1)	28 (38.9)	11 (15.3)	2 (2.8)	72 (100)
Factor 3: Belongingness	5 (6.9)	18 (25.0)	22 (30.6)	7 (9.7)	20 (27.8)	72 (100)
Factor 4: Easiness	1 (1.4)	21 (29.2)	24 (33.3)	18 (25.0)	8 (11.1)	72 (100)
Factor 5: Interpersonal relationships	30 (41.7)	7 (9.7)	1 (1.4)	11 (15.3)	23 (31.9)	72 (100)
Factor 6: Personal life	20 (27.8)	18 (25.0)	16 (22.2)	6 (8.3)	12 (16.7)	72 (100)

It was observed in factor 1 (working conditions) that most respondents (55.6%) agreed the program had a positive interference, although an expressive percentage (20.8%) stated not having an opinion about it. This was the factor that concentrated most positive opinions.

In regard to factor 2 (approval), the highest percentage (38.9%) showed that the workers did not have an opinion about it, revealing lack of knowledge about the organization's quality program, whereas 43.0% provided a positive evaluation. This factor had the highest percentage of the answer not having an opinion about it.

In factor 3 (belongingness), the answer with the highest percentage (30.6%) was not having an opinion about it, and the other answers together revealed the tendency of workers to provide a negative evaluation (37.5%). This factor ranked second - after factor 5 (interpersonal relationships) - in dividing opinions, and had the highest percentage of very negative answers (27.8%).

Likewise, in factor 4 (easiness), the most expressive number was related to not having an opinion about it (33.3%) and this factor gathered more answers, which revealed the workers' tendency to provide a negative evaluation (36.1%).

In factor 5 (interpersonal relationships), the workers' opinion was divided into extremes: 51.4% of workers provided a positive evaluation and, of those, 41.7% evaluated it very positively, while 47.2% of respondents gave a negative evaluation and 31.9% out of them assessed it very negatively. This factor showed the highest number of very positive answers and very negative answers. It concentrated most of the negative opinions.

The result of evaluating factor 6 (personal life) showed that, although 22.2% of workers answered they did not have an opinion about it, most of them (52.8%) provided a positive evaluation. This factor - after factor 5 (interpersonal relationships) - divided opinions, and had the highest percentage of very positive answers (27.8%).

Except for factor 5 (interpersonal relationships), the percentage of answers demonstrated the workers did not have an opinion about it, showing, as in factor 2 (approval), where this event was observed more significantly, that they did not know the implications of a quality program, neither the quality program of the organization. It was also observed that, in four out of six factors, positive answers were prevalent.

## DISCUSSION

This is a public healthcare organization, a reference in the macroregion, designed to develop quality care, teaching, research and extension activities. However, like other healthcare organizations in Brazil, it faces several challenges, including insufficient funding and issues related to internal and external policies that hinder efficient management of the services.^(8,9)^ Although the organization provides care resources that are not very common in the region and renders relevant work to the population, infrastructure and state-of-the-art pieces of equipment and highly skilled professionals contrast with deficiencies in care processes, which are supported by inefficient support services and disarticulated institutional processes,^*^ (in disagreement with what is required from high complex organizations that demand reliability in their *modus operandi*. This practice addresses some concepts, such as sensitiveness to operations, mainly in processes affecting the patient directly, understanding the reasons that put the patients at risk, and willingness to hear and answer the to the workers' ideas, who know how the processes really work and how the patients are exposed to risks.^(10)^

Aiming at improving managerial methods, the organization decided to implement a quality management system which, in turn, faces operational difficulties, including low priority given to its emergence demands, scarcity of resources and issues of internal and external policies of privileges to some specific sectors/individuals/actions/programs; low autonomy, both in economic matters and in defining specific organizational policies, which reflects lack of credibility in its role as an intermediate between services and strategic level; scarcity of qualification, skilled human resources and work structures; difficulties in sharing the system responsibilities with leaders often limited in terms of managerial competences; difficulty in making workers involved; acting in a context disturbed by political instabilities and, lastly, issues related to resistance to change offered by the new proposal.^**^

Its main strategy and, probably, the most clearly noticed by the players of operational processes is the program of continuous improvement, which foresees the establishment of goals for the sectors, which should be met with the technical support from the quality team and checked with regular periodical evaluations, thus clearly encouraging - in a playful manner - the search for excellence.^***^

The nursing team has great participation in the actions required for implementing the quality programs and it is also affected by them.^(11)^

It is known that dissatisfaction with working conditions (factor 1), in addition to the implications to the worker, also contributes to expensive labor disputes, increased turnover and risk to patients. Moreover, team satisfaction directly affects patients' satisfaction and quality of care delivered.^(12)^

Therefore, it is not possible to think of improvement of service quality without offering adequate production conditions to workers, such as fair and appropriate compensation, working conditions, use and development of capacities; opportunities for growth and safety, social integration in the company; constitutionalism; work and total lifespan, and social relevance of life on work.^(13)^ All these issues tend to be fostered by quality programs. The proposal, maybe pretentious to be compatible with the current company management, and can be considered a goal to be reached.

In a qualitative study developed at the same hospital, the nurses reported that implementing the program organized the service and led to safe work by the team; furthermore, while optimizing the processes, it allowed more time to systematize care.^(14)^

Although it is not directly included in the work conditions, it is observed that part of the workers' need is related to concern about the results care due to work process. Positive changes in quality of care were found in healthcare organizations that have implemented quality programs.^(15)^

In approval (factor 2), unawareness of workers about the program was observed. Approval is important for them to be motivated to engage in the actions proposed. It is important to have everyone participating for quality to take place, because this depends on individual and collective efforts.^(16)^ Some studies indicate that few professionals actually understand the meaning of quality management, but they believe it is a tool that is able to intervene in the work process and provide safety. They also report that many workers believe that quality management is under responsibility of quality management sector, and do not realize they are in charge of making this policy effective. Yet, others are waiting for a sudden change arising from the introduction of a quality program, not understanding the process as a daily construction work^(14)^.

Despite the consensus that everyone should strive to do the best, we must also think that everyone should know what to do, which leads to the reflection that is not expected that someone becomes engaged, agrees and consents to what he/she does not understand.^(15)^ The history of program implementation revealed that quality management team engaged in various training activities without effective participation of workers, but it is necessary to consider other strategies to engage them.

Understanding the importance of empowering people in the organization has given rise to participatory learning strategies that, in addition to considering men as mere spectators who must follow orders, they seek to mobilize the creative potential of workers, turning them into part of the learning process through reflection on practice^(17)^.

As for belongingness (factor 3), it can be stated that both quality of work and quality of life at work depend on issues related to the worker's routine, and family and social relationships, including the feeling of belongingness.^(18)^ The result obtained is related to the approval (factor 2), since, by failing to understand, know and engage with the program, the worker does not feel part of it. In the abovementioned study, part of the interviewees acknowledged the importance of taking part in the program, but admitted that they did not take part in it.^(14)^

The results of the American accreditation program for excellence in nursing care entitled Magnet Recognition Program^®^, which brings standards related to leadership, improvement of structure, principles for professional practice, and improvements, new knowledge and innovations to practice and quality results demonstrated that the nursing staff of accredited services report more opportunity to have their opinion accepted on issues related to the organization of work space, participation in shared leadership and an environment with a positive atmosphere.^(19)^

Regarding easiness (factor 4), some studies showed that professionals did not have a clear concept of stress, but they considered experiencing stress related to factors intrinsic to work, relationships, stressful roles and organizational structure.^(20)^

The quality programs by proposing greater control of work processes, can act as stressors in all these dimensions. A study that dealt with the implications of the accreditation process for nursing staff, in addition to stress by the program itself, pointed an unequal demand in regard to following standards and quality care, in relation to the different professional categories in an organization.^(11)^

Additionally, the studies indicated that quality management increases responsibility of workers in production, and often without adequate counterpart.^(18)^ However, the qualitative study conducted at the organization when addressing the difficulties perceived by nurses in implementing the quality program showed that workers felt more under pressure due to difficulties in professional practice arising from organizational problems, rather than because of quality program control.^(14)^

Applying the theory of two factors developed by Herzberg,^(21)^ one can see that environmental or hygiene factors (working conditions, type of supervision, administrative policies, status and prestige, interpersonal relationships, money and personal safety) are the factors that influence in satisfaction of these workers, as shown in other studies on work motivation of the nursing team.^(22)^ Herzberg believes that any change or improvement in these elements will decrease dissatisfaction, but will not increase satisfaction.^(21)^

With respect to interpersonal relationships (factor 5), it should be noted that, adjusting the Deming's quality principles to health, it is shown in all of them the need of the worker's responsibility to quality, but equally the responsibility of organization toward the workers, providing them the necessary conditions; therefore, the quality programs should improve the work environment. ^(1)^ This statement coincides with the opinion of workers who provided a good evaluation for the factor.

Nevertheless, some authors consider that the movement Total Quality, which considers itself as humanistic, democratic and participatory, based on what it inherited from the Human Relations School, intends to be superior to the Scientific Organization of Work, proposed by Taylor. Total Quality is similar to Taylor's proposal in many aspects, such as social division of work; disguising the interests play; by means of claiming that the client benefits most; the Cartesian method based on facts and data; emphasis on education as a solution for the world of work; and the pretentious universal applicability. However, it uses the theory in its refined manipulation of labor.^(5)^

Although the workers may not fully comprehend the scope of these statements, those who negatively evaluated the factor demonstrated they do not agree that the quality program can improve the interpersonal relationships, expressing belief in incapacity and/or strategy of the program to perform it.

Concerning the personal life (factor 6), the result shows that the workers believe that quality does not interfere, corroborating the consideration about the easiness finding (factor 4) in which a significant part of workers answered they were under pressure, but such pressure does not seem to be associated to the quality program, but rather to their working conditions.^(14)^

Some issues, such as increased work load and precarious working conditions have been appointed as responsible for physical and emotional damage, as well as suffering in nursing workers routine.^(23)^

Even if these professionals suffer from stress and perceive unequal demands, they think the process provides positive exchange of experiences and greater opportunities in the job market.^(11)^

It is undeniable that the reality experienced by the organization interferes in the implementation of a quality program and in perception of workers about said in program. Nonetheless the results show the need to invest in strategies able to inform workers about the real scope of the program, so that they will engage and make feasible the operation of the quality policy.

As to the history of fights for the social right to universal access to a quality healthcare system, the discussion about any management in health is considered to go beyond the organizational indicators. The need to foster the implementation of quality programs based on improved services delivered to citizens is demonstrated, together with giving more value to the professionals that produce quality services and social-environmental liability, thus benefiting all stakeholders.

## CONCLUSION

A significant number of respondents had no opinion about the issues discussed, which demonstrated they are not aware of the implications of a quality program. However, most workers believe the program interferes in a positive manner in their working conditions; approve the program but do not feel as taking part in it; do not feel at ease to develop their activities – although uneasiness seems to be more related to issues of their work practice than to demands of the program. Moreover, most consider the program positively interferes in interpersonal relations at work, but this matter was the most controversial among all presented; and they do not recognize the program interferes in their personal life.

## Footnotes

* Universidade Federal de Juiz de Fora. Hospital Universitário de Juiz de Fora. Relatório de Gestão: 1994/2006; Relatório de Gestão: 2007/2009; Plano Diretor do Hospital Universitário da UFJF. [Institutional documents].

** Universidade Federal de Juiz de Fora. Hospital Universitário de Juiz de Fora. Relatório do serviço de Gestão da Qualidade; Planejamento estratégico do serviço de Gestão da Qualidade. [Institutional documents]

*** Universidade Federal de Juiz de Fora. Hospital Universitário de Juiz de Fora. Manual do programa Qualidade Nota 100. [Institutional document]
